# Nano-Curcumin Mitigates Doxorubicin-Induced Reproductive Toxicity via Antioxidant, Anti-Apoptosis, and SIRT1-Modulating Effects in Rat Model

**DOI:** 10.3390/toxics13070574

**Published:** 2025-07-08

**Authors:** Noha A. Alshuwayer, Qamraa H. Alqahtani, Marwa H. Hussein, Raeesa Mohammed, Abdulaziz Siyal, Iman H. Hasan

**Affiliations:** 1 Department of Anatomy, College of Medicine, King Saud University, P.O. Box 2925, Riyadh 11461, Saudi Arabia; rmohammad@ksu.edu.sa (R.M.); asiyal@ksu.edu.sa (A.S.); 2 Department of Pharmacology and Toxicology, College of Pharmacy, King Saud University, P.O. Box 22452, Riyadh 11495, Saudi Arabia; ghamad@ksu.edu.sa (Q.H.A.); mhussein3.c@ksu.edu.sa (M.H.H.)

**Keywords:** nanocurcumin, doxorubicin, SIRT1, apoptosis, testicular toxicity

## Abstract

Background: Doxorubicin (DOX) is a potent anti-cancer agent that is widely described in cancer treatment. However, its administration is often limited by its adverse effects, particularly its testicular toxicity, which can induce infertility in male patients. DOX-induced testicular damage is due to oxidative stress, apoptosis, and inflammation. Nanocurcumin (NCR) is a nano-formulated edition of curcumin with a higher therapeutic potential. NCR has demonstrated antioxidant and anti-inflammatory properties. Methods: This study is designed to inspect the potential validity of NCR on DOX-induced testicular damage in male rats. We used thirty-two Wistar albino rats (150–200 g) and divided them into four groups. NCR (80 mg/kg/ dissolved in 1% CMC) was given orally by oral gavage for 14 days. A single dose of DOX (15 mg/kg) (i.p.) was injected on the 7th day of the experiment. Results: DOX treatment reduced the sperm viability and motility rate, cellular antioxidants, and gonadal hormones; it led to higher levels of inflammatory mediators, necrosis, and sloughing in seminiferous tubules. Conversely, NCR treatment significantly alleviated these side effects by improving sperm count/motility and reducing sperm abnormalities. The testicular function recovery was likely driven by stimulating the cytoprotective SIRT1/NF-κB pathway, depressing the testicular level of oxidative indicators such as MDA, TNF-α, iNOS, IL-1β, and NO, and increasing levels of antioxidants such as GSH and SOD. In addition, NCR contradicted the apoptotic changes by downregulating the pro-apoptotic signals Bax and caspase-3, while inducing Bcl-2 upregulation. Moreover, NCR increased levels of gonadal hormones, attenuated histological abnormalities, and preserved testicular structure when compared with the DOX group. Conclusions: NCR treatment can effectively ameliorate DOX-induced testicular toxicity.

## 1. Introduction

Doxorubicin (DOX) is a powerful anthracycline chemotherapy drug widely utilized to treat various types of cancer [1]. Despite its effectiveness, its clinical use is commonly restricted due to the induction of multiple organ toxicities. In the testes, it leads to hormonal disruptions and infertility [2]. Several preclinical studies have extensively documented DOX testicular toxicity [3], which is revealed as reduced testicular mass, histopathological changes, oligospermia, and abnormal sperm morphology [4]. Mechanistically, DOX increases the reactive oxygen species (ROS) levels and reduces antioxidant enzyme activity, such as SOD and CAT, causing lipid peroxidation and mitochondrial impairment [4]. In addition, it causes the degeneration of seminiferous tubules and loss of germ cells [5]. The drug suppresses steroidogenesis, leading to disruptions in hormonal balance such as reduced testosterone levels and altered FSH/LH concentrations [6]. Furthermore, it activates apoptotic pathways involving p53, Bax, and caspase signaling [7]. However, the exact mechanism of DOX-induced testicular toxicity still needs to be determined.

Curcumin, a polyphenolic compound obtained from *Curcuma longa*, exhibits remarkable anti-inflammatory, antioxidant, and anti-apoptotic properties. Despite these benefits, its clinical potential is thwarted by low bioavailability. Advances in nanocurcumin formulation enhanced their bioavailability. Nano-formulations boosted water solubility and cellular uptake, enhanced tissue distribution and bioavailability. Liposomal formulations, composed of phospholipid vesicles, improve the solubility of curcumin and shield it from degradation within the gastrointestinal tract [8]. Research indicates that liposomal NCR extends its existence in systemic circulation and elevates its plasma bioavailability [9]. Furthermore, liposomes enhance intracellular delivery by promoting membrane fusion or triggering endocytosis, thus improving the uptake of encapsulated curcumin. This mechanism allows for increased therapeutic potency, even at reduced dosages [10]. Due to their sustained release formula, their therapeutic action is prolonged, thus increasing their therapeutic effects [11].

Nanocurcumin has been shown to exhibit antioxidant activities through enhancing the activation of intrinsic antioxidant enzymes, such as superoxide dismutase (SOD) and catalase, while simultaneously lowering malondialdehyde (MDA) levels, effectively mitigating lipid peroxidation in testicular tissue [12]. It also has anti-inflammatory effects, as it inhibits NF-κB activation and decreases pro-inflammatory cytokines levels such as Tumor necrosis factor-α (TNF-α), interleukin-6 (IL-6), and IL-1β. Thus, it may play a role in reducing testicular inflammation [13]. Moreover, NCR suppresses the mitochondrial apoptotic cascade by downregulating Bax and caspase-3 and upregulating Bcl-2, which influences germ cell survival and seminiferous tubule integrity [14]. NCR has the ability to support Leydig cell function and restore normal testosterone levels [15].

PCNA is a nuclear protein that acts as an essential cofactor for DNA polymerase δ, facilitating DNA replication, repair, and progression through the cell cycle [16]. It is indispensable for the proliferation of spermatogonia, primary spermatocytes, and Sertoli cells [17]. DOX-induced testicular toxicity can significantly suppress PCNA expression [18]. Antioxidant therapies, like nanocurcumin, can induce the restoration of its normal levels, which typically reflects testicular recovery [16].

Sirtuin 1 (SIRT1) is a known nicotinamide adenosine dinucleotide (NAD)-dependent deacetylase, which regulates metabolism and maintains homeostasis. SIRT1 can regulate many transcription factors and proteins, e.g., NF-κB, p53, eNOS, PGC-1α, and AMPK [19].

SIRT1 expression and activity are substantially decreased in DOX-induced cardiotoxicity. Enhancing its expression can alleviate cellular injury through different pathways, via defending against oxidative stress, mitochondrial dysfunction, and apoptosis [20]. SIRT1’s antioxidant activities play a vital role in the male reproductive system through its participation in spermatogenesis [21]. SIRT1 is abundantly present in spermatogonia, spermatocytes, and round spermatids. It plays a critical role in maintaining the equilibrium between the self-renewal and differentiation of spermatogonial stem cells (SSCs) [22]. Additionally, it influences chromatin restructuring and histone deacetylation, processes vital for accurate meiotic progression and the maturation of sperm cells [22]. SIRT1 boosts steroidogenic function in Leydig cells by deacetylating and activating key transcription factors, such as SF-1 and CYP11A1, which are crucial for driving testosterone biosynthesis [23] and limiting oxidative stress within Leydig cells, thus maintaining their structural integrity and hormone-producing capacity [22]. In testicular tissues, SIRT1 activation inhibits p53-mediated apoptosis, suppresses NF-κB signaling, and enhances mitochondrial biogenesis via PGC-1α [24,25].

SIRT1 inhibition of NF-κB leads to the downregulation of TNF-α, IL-1β, IL-6, and blocks COX-2 and iNOS expression [26].

Recently, NCR has been found to activate the SIRT1 pathway, an essential regulator of cellular stress endurance and metabolism in brain tissues [25]. Curcumin augments SIRT1-driven DNA repair by upregulating key repair proteins and attenuating oxidative DNA lesions [27]. This action promotes genomic integrity and supports the restoration of spermatogenesis, particularly in DOX-induced testicular toxicity. The SIRT1/NF-κB signaling pathway is essential for developing the antioxidant function of testicular tissues and lowering oxidative stress [28].

We believe that using NCR instead of curcumin offers a multifaceted protective alternative against doxorubicin-induced testicular toxicity through its antioxidant and anti-inflammatory defense mechanisms, due to its activation of the SIRT1 cytoprotective signaling pathway, along with its hormonal regulatory properties.

## 2. Materials and Methods

### 2.1. Drugs and Chemicals

The chemotherapeutic agent (DOX) used to induce testicular damage was acquired from Sigma (St. Louis, MO, USA). Liposomal nanocurcumin (NCR) was supplied by Lipolife (Essex, UK). The testosterone ELISA kit was obtained from Cusabio (Wuhan, China). TNF-α, interleukin-1β, SIRT1- ELISA kits were provided by R&D Systems (Minneapolis, MN, USA), and FSH and LH ELISA kits and antibodies against iNOS, NF-KB-P65, and PCNA were provided by Novus Biologicals (Centennial, CO, USA). Other chemicals used for oxidative stress, histopathological examination and carboxymethylcellulose (CMC) were acquired from Sigma (St. Louis, MO, USA).

### 2.2. Animals and Experimental Design

Thirty-two adult male Wistar albino rats (150–200 g) were taken from the Bio-Resource Unit at the College of Pharmacy, King Saud University, Saudi Arabia. The rats were housed at a standard temperature and humidity with a 12 h light/dark cycle, and they had free access to food and water. The experimental design was established in agreement with the recommendations of the Institutional Animal Care and Use Committee (IACUC); the experimental protocol was approved by the Research Ethics Committee at King Saud University (KSU-SE-24-68). Rats were randomly divided into four groups (8 rats/group) as follows: Group I (Control) rats were given the vehicle solution (1% CMC) for 14 days and were injected with saline on the 7th day i.p.; Group II (NCR) rats were given NCR (80 mg/kg dissolved in 1% CMC) orally by oral gavage for 14 days [15]. Group III (DOX) rats were given vehicle solutions orally for 14 days and were injected with a single dose of DOX (15 mg/kg, i.p.) on the 7th day of the study [29]; Group IV (DOX+NCR) rats were given NCR (80 mg/kg BW in normal saline) orally by oral gavage for 14 days [16], seven days before and after DOX injection (15 mg/kg, i.p.) on the 7th day of the experiment.

Twenty-four hours post-injection (on day 15), the rats were anesthetized using carbon dioxide (CO_2_), sacrificed by decapitation, and blood and testicular samples were immediately collected. Centrifugation of blood samples was conducted at 3000 rpm at 4 °C for 30 min to obtain clear sera, which were then stored at −80 °C. Sperm samples were collected (on slides) for sperm morphology and physiological examination. The testicles and epididymides were carefully separated from surrounding tissue adhesion and washed in cold phosphate-buffered saline (PBS). Testicular tissue parts were fixed in 10% formalin solution, while homogenization of other samples was obtained (10% *w*/*v*) using Tris–HCl buffer (pH 7.4). Centrifugation of the homogenate and collection of the supernatant were conducted to determine MDA, nitric oxide (NO), (GSH), SOD, TNF-α and IL-1β. Additional tissues and blood samples were cryopreserved at −80 °C for further analysis.

### 2.3. Sperm Count, Viability, and Morphology

At the end of the experiment, after the rats were euthanized, the cauda epididymis was extracted and sliced. Subsequently, it was immersed in 2 mL phosphate-buffered saline (PBS) at a pH of 7.4, maintained at 37 °C. The sperm suspension was diluted with PBS (pH 7.2) at a 1:20 ratio. The total sperm count was revealed using a hemocytometer. Sperm viability and the total number were counted in five squares and multiplied by 10^6^ to obtain the final volume [30] (Omar et al., 2022). In addition, epididymal sperm morphology was assessed by staining a smear using hematoxylin and eosin (H and E) and microscopic examination under a light microscope at 1000X.

### 2.4. Assay of Testosterone, Gonadotropin Hormones, and Inflammatory Biomarkers

Serum levels of testosterone hormone, FSH, and LH levels were measured. Simultaneously, ELISA kits were used to evaluate IL-1β, TNF-α, and SIRT1in the testicular tissue supernatant, according to the manufacturer’s instructions.

### 2.5. Assay of Antioxidants

Testicular malondialdehyde lipid peroxidation, assessed as MDA using the methods of Preuss et al., along with levels of reduced GSH, following Beutler et al., were measured in the testicular homogenate [31,32]. The antioxidant activity of the SOD enzyme was determined according to the protocols of Marklund and Marklund [33]. The testicular tissue supernatant was used to assay the NO level following the method of Grisham et al. [34].

### 2.6. Gene Expression

The gene expressions of B cell lymphoma-2 (BCL-2), BCL-2-associated X protein (Bax), and caspase-3 were estimated through qRT-PCR. TRIzol (ThermoFisher Scientific, Waltham, MA, USA) was employed to isolate RNA, which was treated with RNase-free DNase and quantified on a nanodrop. β-actin was employed as the housekeeping gene to normalize the expression levels. RNA samples with an A260/A280 nm ratio of 1.8–2.0 were selected for cDNA synthesis and amplified using the primers in Table 1 and Maxima SYBR Green/ROX qPCR master mix (ThermoFisher Scientific, Waltham, MA, USA). Data were evaluated using the 2 _∆∆Ct method [35] with normalization to β-actin.

### 2.7. Histopathological and Immunohistochemistry (IHC) Examination

The testicular samples were fixed for 24 h in 10% formalin in normal saline and then handled through the standard paraffin procedure. The tissues were sliced into 5-µm sections, then stained with H and E. To estimate spermatogenesis, the light microscope was used for visualization. Afterwards, Johnsen’s criteria were used to grade spermatogenesis. According to the presence or absence of the primary cell types, preservation of spermatogenesis was graded from 1 to 10. A Johnsen score of 9 or 10 specifies normal histology; a score of 8 indicates hypospermatogenesis; a score of 3–7 denotes maturation arrest; a score of 2 implies germinal cell aplasia; and a score of 1 indicates tubular fibrosis. The germinal epithelium of the tubules was evaluated for each separate testes. Then, the average score was estimated for each single rat. Similarly, areas and volumes of seminiferous tubules in the testes were measured.

The IHC analysis was used to examine the changes in the immunoreactivity of testicular iNOS, and PCNA, expression. The slices were dewaxed; antigen retrieval was achieved by immersion in 0.05 M citrate buffer (pH 6.8). The slides were probed with diluted anti-iNOS and PCNA (sc-7271; sc-56, respectively, Santa Cruz Biotechnology, Inc., Dallas, TX, USA) (1:100) overnight at 4 °C after treating them with 0.3% hydrogen peroxide (H_2_O_2)_ and protein block. The slides were washed in PBS and a secondary antibody was placed for 60 min at 25 °C. Lastly, a DAB kit was used to obtain the color.

### 2.8. Statistical Analysis

The results are stated as the mean ± standard error of the mean (SEM). One-way ANOVA was used, followed by Tukey’s post hoc test for multiple comparison after applying the Shapiro–Wilk test, and confirmed the homogeneity of variances using Levene’s test. GraphPad Prism 9 was used for statistical analysis. The data were considered statistically significant if the *p* value < 0.05.

## 3. Results

### 3.1. NCR Significantly Mitigates Decreased Body Weight Caused by DOX, and Neither Affect Testicular Weight

The results showed that the group treated with NCR alone had outcomes comparable to the control group across all physical parameters, like body or testis weight, indicating that NCR treatment does not negatively impact testicular function. DOX-treated rats exhibited a significant decrease in body weight change when compared to the control group (*p* < 0.001). However, DOX+NCR significantly mitigated body weight change in comparison to the DOX group (*p* < 0.01). Furthermore, no significant change in testis weight was noted between all the treatment groups (Table 2).

### 3.2. NCR Attenuates the Effect of DOX on Sperm Morphology, Count, Abnormality, and Rapid Progressive Mobility

**Figure 1:**
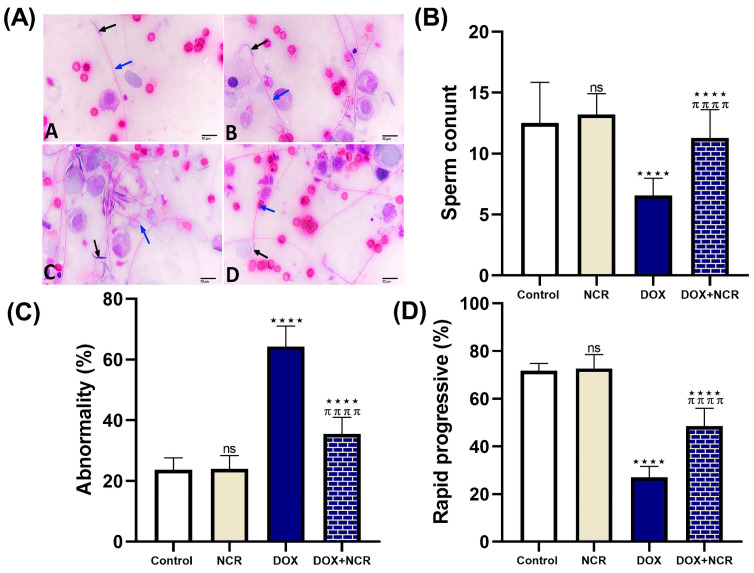
Effects of NCR treatments on: (**A**) sperm morphology as observed under light microscopy (magnification: ×400); (**B**) sperm count, total number of sperm cells in the epididymal suspension; (**C**) sperm abnormality ratio—calculated based on morphological defects such as head deformities (black arrow); midpiece anomalies, and tail defects (blue arrow); and (**D**) rapid progressive ratio—proportion of sperm showing fast forward progression—against testicular toxicity of DOX in the rat model. Data are expressed as mean ± SD (n = 8/group); (ns; non-significant, **** *p* ≤ 0.001 vs. control group, and ^π π π π^ *p* ≤ 0.001 vs. DOX group).

The represented results for sperm morphology (Figure 1A) show that the effects of DOX on the sperm shape were attenuated after NCR was administrated. The sperm count examination (Figure 1B) shows that DOX administration significantly reduced sperm count, by 59.4%, in comparison to the control. In contrast, a significant increase in sperm count in the DOX+NCR-treated group, of 48.9%, was observed when compared with the DOX-treated group. The sperm abnormality ratio analysis (Figure 1C) shows that DOX caused a significant increase in sperm abnormality (64.3 ± 2.7 vs. 23.7 ± 1.6, *p* ≤ 0.001) compared to the control group. However, the DOX+NCR-treated group showed a lower decrease in sperm abnormality than the DOX group (35.5 ± 2.2 vs. 64.3 ± 2.7, *p* ≤ 0.001). Figure 1D shows the sperm mobility ratio in all groups; statistical analysis of the data showed that there was a significant decrease in the sperm rapid progressive ratio in the DOX group in comparison to the control group (27 ± 1.9 vs. 71.7 ± 1.28, *p* ≤ 0.001), while there was a significant increase in DOX+NCR as compared to the DOX-administrated group (48.5 ± 3.3 vs. 27 ± 1.9, *p* ≤ 0.001). As is evident from the results shown in Figure 1A–D, there is no clear difference in the physical parameters for sperm compared to NCR alone and the control group.

### 3.3. NCR Modulates the Effect of DOX on Seminiferous Tubules—Normal; Detached and Sloughed; And Vacuolated Histology in Testicular Tissue

Our data in Table 3 present the histological evaluation of seminiferous tubules in testicular sections across the different experimental groups. Tubules were categorized based on morphological integrity into three types: normal; detached and sloughed (indicating the loss of germ cells from the epithelium); and vacuolated (indicating cytoplasmic or intercellular vacuolation). The percentages reflect the proportion of tubules in each category, providing insight into testicular damage or preservation associated with each treatment. A higher percentage of normal tubules indicates better preservation of testicular structure, while increased percentages of sloughed or vacuolated tubules suggest testicular injury or degeneration.

### 3.4. NCR Effects on DOX-Induced Testicular Histopathological Changes

The histopathological analysis of the testicular tissue, the control group, and the NCR-treated group showed no noticeable histopathological alterations. The seminiferous tubules in both groups maintained normal structure and cellular organization, indicating that NCR alone does not exert any harmful effects on testicular tissue. The DOX group revealed significant structural damage. These changes included disorganized seminiferous tubules, degeneration of germinal epithelium, exfoliation of spermatogenic cells, vacuolation, and reduced spermatogenesis. These findings are consistent with DOX-induced testicular toxicity, likely due to inflammation, oxidative stress, and apoptosis triggered by the chemotherapeutic agent.

**Figure 2:**
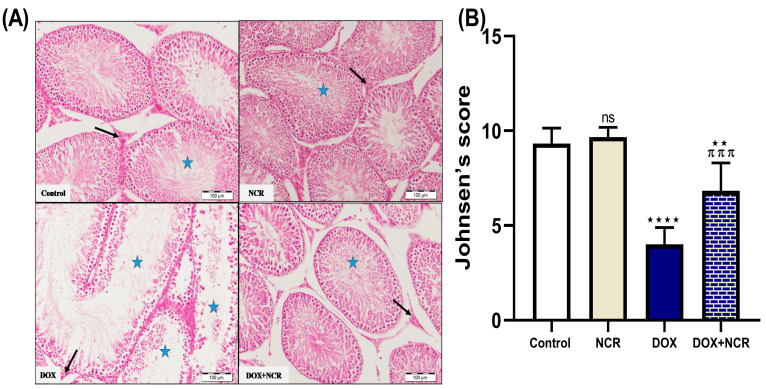
(**A**) Sections of rat testis stained with H and E. Scale bar 100 µm. Normal control rat and rat receiving NCR, both showing normal seminiferous tubules (stars), and in-between interstitial tissue (arrows); while testicular tissue from rat receiving DOX shows disappearance of stratified spermatogenic cells of most of seminiferous tubules (stars), and shrinkage of interstitial tissue containing Leydig cells (arrow). Testis tissue in DOX+NCR group shows marked regeneration of seminiferous tubule integrity (star) and prominent interstitial tissue in-between (arrow). (**B**) Johnsen’s spermatogenesis score. Data are expressed as mean ± SD (n = 8/group). (ns; nonsignificant, ** *p* ≤ 0.05, **** *p* ≤ 0.001 vs. control group, and ^π π π^ *p* ≤ 0.001 vs. DOX group).

In contrast, co-treatment with NCR significantly alleviated these histopathological alterations. The NCR-treated group exhibited improved seminiferous tubule architecture, preservation of germinal epithelium, and reduced cellular degeneration and vacuolation, in addition to improved spermatogenic activity. Notably, the group treated with NCR alone showed no noticeable histopathological alterations in comparison to the control group. The seminiferous tubules in both groups maintained normal structure and cellular organization, indicating that NCR alone does not exert any harmful effects on testicular tissue (Figure 2A).

Furthermore, the changes in spermatogenesis between the different groups was estimated using Johnsen’s score. DOX-intoxicated rats indicated a marked decline in Johnsen’s score compared to the control and NCR-treated groups. Additionally, a substantial increment in such scores was observed in the testes of the DOX+NCR-treated rats compared with the DOX-intoxicated group. Furthermore, the NCR group demonstrated a significant advancement in Johnsen’s score prior to that of the control group (Figure 2B).

### 3.5. NCR Attenuates the Effect of DOX on Reproductive Hormones

**Figure 3:**
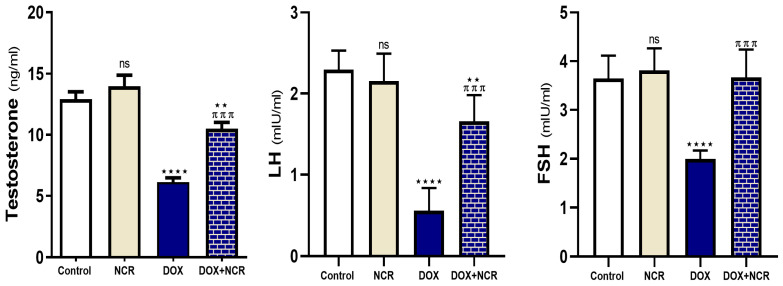
Effects of NCR treatments on serum reproductive hormones in testicular toxicity induced by DOX in the rat model. Data are expressed as mean ± SD (n = 8/group) (ns; nonsignificant, ** *p* ≤ 0.05, **** *p* ≤ 0.001 vs. control group, and ^π π π^ *p* ≤ 0.001 vs. DOX group).

The results in Figure 3 illustrate the impact of NCR on key reproductive hormone levels, including testosterone, LH, and FSH. DOX administration led to a significant reduction in serum testosterone levels (6.13 ± 0.3 vs. 12.9 ± 0.6, *p* ≤ 0.001), likely due to its known gonadotoxic effects that impair Leydig cell function. Additionally, DOX treatment may have caused disruptions in the hypothalamic–pituitary–gonadal (HPG) axis, reflected by a significant elevation in LH (0.56 ± 0.1 vs. 2.3 ± 0.1, *p* ≤ 0.001) and FSH (1.99 ± 0.07 vs 3.65 ± 0.2 *p* ≤ 0.001) levels in comparison to the control group. Conversely, co-administration of NCR appeared to mitigate the adverse effects of DOX on reproductive hormones. Groups receiving NCR alongside DOX showed partially restored levels of testosterone (10.5 ± 0.5 vs. 6.13 ± 0.3, *p* ≤ 0.001), LH (1.7 ± 0.1 vs. 0.56 ± 0.1, *p* ≤ 0.001), and FSH (3.67 ± 0.23 vs. 1.99 ± 0.07, *p* ≤ 0.001) compared to the DOX group. This suggests a protective role of NCR, possibly due to its anti-inflammatory and antioxidant activities, which may help to preserve testicular function and endocrine balance.

### 3.6. NCR Attenuated the Oxidative Stress and Inflammatory Biomarkers in Testicular Tissues in Rats Treated with DOX

**Figure 4:**
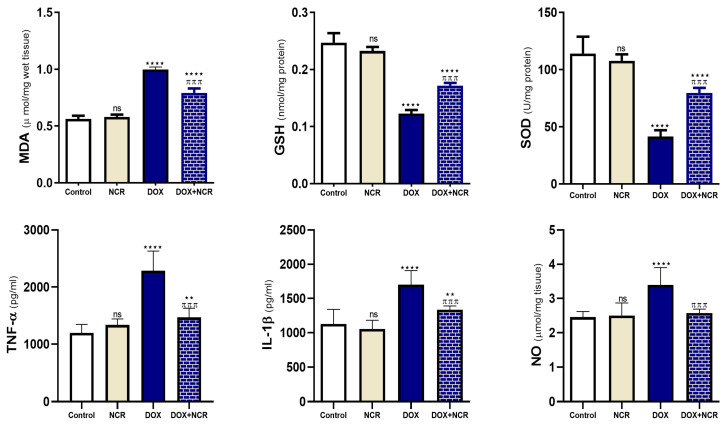
NCR reduces testicular oxidative stress and inflammatory biomarkers in DOX-intoxicated rats. Treating rats with NCR decreased MDA and NO, TNF-α, and IL-1β; moreover, NCR administration increased GSH level and SOD activity. Data are expressed as mean ± SD (n = 8/group) (ns; nonsignificant, ** *p* ≤ 0.05, **** *p* ≤ 0.001 vs. control group, and ^π π π^ *p* ≤ 0.001 vs. DOX group).

The biochemical analysis of testicular tissue demonstrated that treatment with DOX significantly increased oxidative stress (Figure 4), as evidenced by elevated levels of MDA, a key marker of lipid peroxidation (0.99 ± 0.02 vs. 0.56 ± 0.02 μmol/mg, *p* ≤ 0.001). Additionally, DOX-treated rats exhibited marked reductions in the activities of SOD (41.6 ± 5.5 vs. 114 ± 14.8 U/mg protein, *p* ≤ 0.001), GSH (0.13 ± 0.00 vs. 0.25 ± 0.01 nmol/mg protein, *p* ≤ 0.001), and NO (3.39 ± 0.02 vs. 2.46 ± 0.06 μmol/mg, *p* ≤ 0.01), indicating impaired antioxidant defense mechanisms. Conversely, rats co-treated with NCR showed a remarkable attenuation of DOX-induced oxidative responses. NCR significantly reduced MDA levels (0.79 ± 0.03 vs. 0.99 ± 0.02 μmol/mg, *p* ≤ 0.01), while restoring the activities of SOD (79.8 ± 4.3 vs. 41.6 ± 5.5 U/mg protein, *p* ≤ 0.001), and GSH (0.17 ± 0.00 vs. 0.13 ± 0.00 nmol/mg protein, *p* ≤ 0.05) and NO (2.57 ± 0.07 vs. 3.39 ± 0.02 μmol/mg, *p* ≤ 0.01) towards normal values. These changes indicate a reduction in oxidative damage and enhancement of the antioxidant defense system.

Furthermore, DOX administration led to a significant rise in inflammatory biomarkers (Figure 4), particularly TNF-α (2287 ± 140 vs. 1197 ± 60.6 pg/ml, *p* ≤ 0.001) and IL-1β (1701 ± 83.7 vs. 1129 ± 86.2 pg/ml, *p* ≤ 0.001), suggesting the activation of inflammatory pathways in testicular tissue. Additionally, NCR treatment led to a significant decrease in TNF-α (1467 ± 65.3 vs. 2287 ± 140 pg/ml, *p* ≤ 0.001) and IL-1β (1334 ± 23.7 vs. 1701 ± 83.7 pg/ml, *p* ≤ 0.001) levels in comparison to the DOX group, suggesting that NCR effectively suppressed the inflammatory response associated with DOX toxicity. Overall, these results prove that NCR exerts a strong protective effect against DOX-induced oxidative stress and inflammation in testicular tissues, likely because of its potent antioxidant and anti-inflammatory activities.

### 3.7. NCR Attenuated the Caspase-3, Bax, Bcl2 Gene Expression, and SIRT1 Level in DOX-Testicular Toxicity

**Figure 5:**
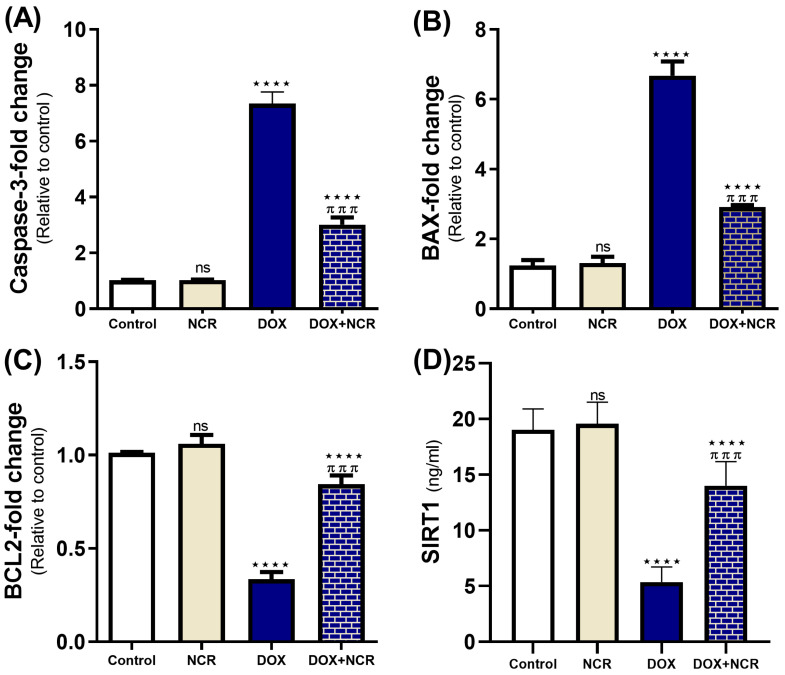
Nano-curcumin (NCR) modulates the expression of apoptosis-related genes in the testes of doxorubicin (DOX)-treated rats: (**A**–**C**) NCR significantly downregulated the mRNA expression of caspase-3 and Bax, while upregulating BCL-2 expression; (**D**) NCR also enhanced the expression level of SIRT1 in testicular tissue. Data are expressed as mean ± SD (n = 8/group) (ns; nonsignificant, **** *p* ≤ 0.001 vs. control group, and ^π π π^ *p* ≤ 0.001 vs. DOX group).

Figure 5 shows that treatment with NCR significantly attenuated the reduction in the gene expression of key apoptotic and survival markers induced by DOX, specifically, caspase-3, BCL2, and Bax, which were qRT-PCR evaluated. In the DOX-treated group, caspase-3 (7.352 ± 0.4 vs. 1.01 ± 0.00-fold change to control, *p* ≤ 0.001) and Bax (6.68 ± 0.4 vs. 1.24 ± 0.2-fold change to control, *p* ≤ 0.001) expressions were significantly elevated, consistent with the induction of apoptosis. On the other hand, downregulation of BCL2 (0.34 ± 0.03 vs. 1.01 ± 0.00-fold change to control, *p* ≤ 0.001) occurred. However, in the NCR +DOX co-treated group, caspase-3 expression levels were significantly reduced compared to DOX (3.01 ± 0.26 vs. 7.352 ± 0.4-fold change to control, *p* ≤ 0.001), indicating that NCR mitigated the apoptotic signaling also by decreasing Bax expression (2.9 ± 0.05 vs. 6.68 ± 0.4-fold change to control, *p* ≤ 0.001). In addition, a restoration of BCL2 expression (0.84 ± 0.04 vs. 0.34 ± 0.03-fold change to control, *p* ≤ 0.001) in the NCR-treated group was found compared to the DOX-only group (Figure 5).

Additionally, the tissue levels of SIRT1, a key regulator of cellular stress responses, were markedly reduced following DOX treatment (5.36 ± 0.6 vs. 19 ± 0.7 ng/ml, *p* ≤ 0.001). However, NCR co-treatment reversed this decline, significantly enhancing SIRT1 levels compared to the DOX group (14 ± 0.9 vs. 5.36 ± 0.6 ng/ml, *p* ≤ 0.001). These results imply that NCR exert a protective effect against DOX-induced cellular damage by modulating apoptotic pathways and enhancing cellular survival mechanisms (Figure 5).

### 3.8. NCR Attenuated the DOX-Induced Change in iNOS and PCNA Expression

**Figure 6:**
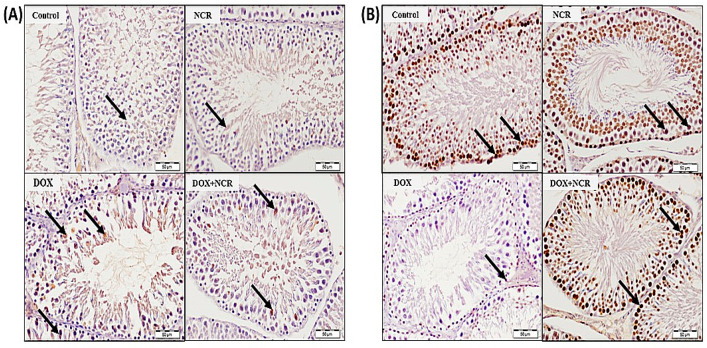
(**A**) Sections of rat testis immunostained with iNOS primary antibody; the normal group and rats receiving NCR showed very weak positive reactions (arrows). Rats receiving DOX showed strong positive immune reaction of spermatogenic cells of seminiferous tubules (arrows), while rats administrated with NCR and receiving DOX showed a marked decrease in iNOS immune positivity (arrows); and (**B**) sections of rat testes immunostained with PCNA primary antibody; testes tissues from normal control rats and rats receiving NCR show strong positive reactions (arrows). On the other hand, there was a very weak immune reaction of spermatogenic cells of seminiferous tubules of testes from rats receiving DOX (arrow), finally rats in the DOX+NCR group showed a marked increase in immune positivity (arrows) (Scale bar 50 µm).

The protein levels of iNOS, and PCNA were inspected by immunopositivity reaction in testes tissues. The administration of DOX triggered upregulation of testicular iNOS protein levels (Figure 6A) and downregulated PCNA (Figure 6B) in comparison to the control rats (Figure 6A,B).

## 4. Discussion

Doxorubicin (DOX) is widely used as an anti-cancer agent; however, its prescription is constrained due to severe side effects, particularly concerning male reproductive health [18] One major issue is DOX-induced testicular toxicity, which significantly impacts sperm quality, causes testicular atrophy, and leads to infertility. These effects not only compromise fertility but also diminish the whole life quality of male cancer patients. The primary mechanisms behind this toxicity involve oxidative stress, inflammation, DNA damage, and apoptosis [6]. The present study aimed to investigate the possible modulatory effect of NCR against testicular toxicity induced by DOX and the potential underlying mechanisms. Based on our study findings, NCR, an improved curcumin formulation, shows great promise as a therapeutic approach. These effects most probably occur due to its strong antioxidant and anti-inflammatory effects. NCR helps to counteract DOX-induced damage to testicular tissue more effectively than traditional curcumin.

Our study results have shown that DOX increased percentage changes in sperm abnormalities as well as decreased sperm count and sperm motility. These results align with Belhan, et al., 2020, who justified these changes by the enhanced ROS generation, increased oxidative stress, DNA fragmentation, and chromosomal aberrations induced by DOX in male albino Wistar rats [36]. In contrast, NCR significantly reduced malformed sperm forms, concomitant with a significant enhancement in sperm count and motility. These improvements agree with a previous study, which testified that curcumin markedly enhanced sperm motility, viability, and sperm count in Wistar rats, following a lead acetate treatment [37]. These protective effects occur due to the antioxidant activity of NCR. Similar results were seen in rats when using other antioxidants, as mentioned in this study; the antioxidant compounds in the leaves of *S. anomalum* attenuated DOX-induced toxicity [38].

In this study, the histopathological examination revealed that DOX induced significant structural damage, including disorganized seminiferous tubules, exfoliation of the spermatogenic cell degeneration of germinal epithelium, vacuolation, and reduced spermatogenesis. These findings are likely due to oxidative stress, inflammation, and apoptotic effects. Comparable alterations due to DOX-induced testicular toxicity have been formerly stated. Abdelaziz et al. 2019 proved that treatment with DOX in prepubertal rats led to testicular tissue atrophy, decreased germ cell density, and caused thinning in seminiferous tubule walls [39]. However, our study results showed that NCR enhanced seminiferous tubule architecture, decreased cellular degeneration and vacuolation, boosted spermatogenic activity, and protected germinal epithelium.

In the current study, DOX-induced testicular toxicity caused a significant decline in testosterone hormone levels in blood. These findings agree with a preceding study conducted on Wistar rats that reported a reduction in testosterone levels induced by DOX [40]. This effect is probably attributed to the high level of ROS, causing impairment of Leydig cells. Nevertheless, the administration of NCR restored testosterone and FSH to a normal level, mostly due to the reduction in ROS levels. 

DOX-induced oxidative stress is one of the major reasons of testicular damage, leading to lipid peroxidation, DNA fragmentation, and cellular apoptosis. Dox administration caused a significant increase in the testicular MDA level while inducing a significant decline in testicular SOD and GSH levels. These results are consistent with those of Asanga et al. 2024 [38]. However, co-treatment with NCR enhanced the levels of these antioxidants, illuminating its antioxidative activity. The ability of NCR to counteract oxidative damage has been well-documented in various studies. Several studies have reported that NCR significantly increases the level of antioxidant enzymes, such as SOD and GSH, while reducing the MDA level. In a study by Salimi et al. (2022), NCR demonstrated heightened antioxidant effects compared to regular curcumin, providing greater protection against oxidative damage in hepatic tissues, including MDA [40]. In addition, another study demonstrated that the administration of NCR protected the kidney and liver against oxidative stress, as verified by a significant increase in antioxidant activities of SOD and GSH [41].

The SIRT1 signaling pathway engages with a variety of other cellular pathways, such as NF-κB, Nrf2/HO-1, and mTOR, thereby helping to control inflammation and oxidative stress [42]. Through SIRT1/Nrf2/HO-1 in the testes, SIRT1 deacetylates Nrf2 to enhance the transcription of genes involved in antioxidant defense such as HO-1 and GPx. These actions collectively lead to protecting Sertoli and Leydig cells against oxidative damage, inhibiting lipid peroxidation, and shielding germ cells from DNA injury, in addition to mitigating inflammation and apoptotic processes [43].

Furthermore, oxidative stress in the testicular tissues leads to the activation of NF-κB, which in turn promotes more inflammation and tissue injury. Under such conditions, SIRT1 levels are typically reduced, admitting sustained NF-κB activity. However, enhancing SIRT1 expression has been shown to negatively regulate NF-κB activity, highlighting its role in modulating inflammation [44].

The current study found a significant decline in the protein expression of SIRT1 in DOX-induced toxicity in testicular tissues. In the presence of NCR, SIRT1 gene expression was significantly elevated, resulting in elevated levels of SOD and GSH production with a decrease in TNF-α and IL-6 levels. These results suggest that the SIRT1/NF-κB signaling pathway is fundamental for enhancing the antioxidant capability of testicular tissue and reducing oxidative stress. In many studies, curcumin and NCR have decreased oxidative stress in the brain and other tissues through activation of SIRT1 [45,46].

Different experimental studies have shown that NCR reduces the levels of TNF-α, IL-6, and other pro-inflammatory cytokines, which are upregulated during DOX-induced toxicity [44]. This impact is assigned to the modification of the NF-κB signaling pathway [42]. In this study, DOX induced upregulation in the expression of pro-inflammatory genes, TNF-α and iNOS. These findings are coherent with a previous study of DOX effects on rat testes conducted by Ujah et al. 2021 using real-time RT-qPCR [18]. In our study, NCR was shown to significantly reduce inflammatory markers, protecting the testes from inflammation induced by DOX.

Apoptosis is a significant mechanism by which DOX induces testicular toxicity. DOX upregulates the testicular expression of Bax and caspase-3 and downregulates Bcl2 gene expression [18]. Upregulation of Bax, combined with the downregulation of Bcl-2, disrupts mitochondrial integrity, initiating the activation of caspase-3. This cascade leads to DNA fragmentation, membrane blebbing, and cellular detachment [47]. Conspicuously, caspase-3 activation in germ cells is closely linked to the sloughing of these cells from the seminiferous epithelium [48,49]. In this study, NCR was found to inhibit DOX-induced apoptosis by modulating apoptotic signaling pathways. It prevented the activation of caspases and enhanced the upregulation of Bcl-2, the anti-apoptotic proteins.

In a study conducted by Abdel Fattah et al. 2023, curcumin nanoparticles protected against submandibular salivary gland toxicity induced by methotrexate in male rats [47]. Moreover, nanocurcumin was shown to defend against apoptosis in various organs, including the heart. In a study by Khadrawy et al. (2021), the administration of NCR led to a significant reduction in DOX-induced Bax and caspase-3, as well as an increase in Bcl-2, preserving the integrity of cardiac architecture and function in a mouse model [50]. Furthermore, when assessing the level of PCNA protein using immunohistochemistry, DOX was found to decrease its level in the testes of rats, which is consistent with the results of a previous study [18]. Conversely, NCR was significantly effective in upregulating the expression of PCNA. These results agree with our previous study using NCR against copper-induced testicular toxicity [15].

## 5. Conclusions and Future Directions

Nanocurcumin is believed to be a promising therapeutic adjuvant to alleviate DOX-induced testicular toxicity. Preserving male fertility in cancer patients undergoing DOX treatment can be achieved using NCR due to its improved bioavailability, coupled with its effective antioxidant, anti-apoptotic, and anti-inflammatory activities. While the preclinical results are favorable, additional studies are needed to assess the longstanding protection, ideal dosing, and clinical efficacy of NCR in male cancer survivors facing fertility issues. Future research should also investigate using NCR in combination with other effective agents to accomplish synergistic effects.

## Figures and Tables

**Table 1:** Primers used for qRT-PCR.

Gene	Primers (5′-3′)	Size (bp)
**BCL-2**	F: ACTCTTCAGGGATGGGGTGA R: TGACATCTCCCTGTTGACGC	94
**Bax**	F: AGGACGCATCCACCAAGAAG R: CAGTTGAAGTTGCCGTCTGC	166
**Caspase-3**	F: GGAGCTTGGAACGCGAAGAA R: ACACAAGCCCATTTCAGGGT	169
**β-actin**	F: AGGAGTACGATGAGTCCGGC R: CGCAGCTCAGTAACAGTCCG	1140

**Table 2:** Changes in body weight and testicular weight.

Groups	Initial Body Weight (gm)	Final Body Weight (gm)	Testes Weight/Final B Wt. (g/100 gm B Wt)
**Control**	186 ± 3.7	255 ± 8.7	0.94 ± 0.03
**NCR**	189 ± 2.9	207 ± 16.5	0.95 ± 0.01
**DOX**	196 ± 2.8	167 ± 3.3 ***	0.82 ± 0.04
**DOX+NCR**	192 ± 3.2	205 ± 4.8 ** ^π^	0.88 ± 0.02

Values are expressed as mean ± SD (n = 8/group). (** *p* ≤ 0.01, *** *p* ≤ 0.001 vs. control group, and ^π^ *p* ≤ 0.05 vs. DOX group).

**Table 3:** Percentage of seminiferous tubules exhibiting normal, detached and sloughed, or vacuolated histology in testicular tissue.

Groups	Percentage of Tubules		
	Normal	Detached and Sloughed	Vacuolated
**Control**	84.78 ± 3.8	2.5 ± 0.07	2.67 ± 0.045
**NCR**	92.4 ± 2.09	2.67 ± 0.04	3.02 ± 0.02
**DOX**	12.5 ± 1.56 ****	66.89 ± 4.8 ****	47.7 ± 2.4 ****
**DOX+NCR**	44.6 ± 3.7 ^π π π^ ****	26.8 ± 4.6 ^π π π^ ****	17.7 ± 4.09 ^π π π^ ****

Values are expressed as mean ± SD (n = 8/group. (**** *p* ≤ 0.001 vs. control group, and ^π π π^ *p* ≤ 0.001 vs. DOX group).

## Data Availability

Data generated during this study are available upon request from the corresponding author.
